# *Schisandra chinensis* Peptidoglycan-Assisted Transmembrane Transport of Lignans Uniquely Altered the Pharmacokinetic and Pharmacodynamic Mechanisms in Human HepG2 Cell Model

**DOI:** 10.1371/journal.pone.0085165

**Published:** 2014-01-27

**Authors:** Charng-Cherng Chyau, Yaw-Bee Ker, Chi-Huang Chang, Shiau-Huei Huang, Hui-Er Wang, Chiung-Chi Peng, Robert Y. Peng

**Affiliations:** 1 Research Institute of Biotechnology, Hungkuang University, Shalu County, Taichung City, Taiwan; 2 Department of Food And Applied Technology, Hungkuang University, Shalu County, Taichung City, Taiwan; 3 Graduate Institute of Clinical Medicine, Taipei Medical University, Taipei, Taiwan; 4 Research Institute of Medical Sciences, Taipei Medical University, Taipei, Taiwan; University of Insubria, Italy

## Abstract

*Schisandra chinensis* (Turz Baill) (*S. chinensis*) (SC) fruit is a hepatoprotective herb containing many lignans and a large amount of polysaccharides. A novel polysaccharide (called SC-2) was isolated from SC of MW 841 kDa, which exhibited a protein-to-polysaccharide ratio of 0.4089, and showed a characteristic FTIR spectrum of a peptidoglycan. Powder X-ray diffraction revealed microcrystalline structures within SC-2. SC-2 contained 10 monosaccharides and 15 amino acids (essential amino acids of 78.12%w/w). In a HepG2 cell model, SC-2 was shown by MTT and TUNEL assay to be completely non-cytotoxic. A kinetic analysis and fluorescence-labeling technique revealed no intracellular disposition of SC-2. Combined treatment of lignans with SC-2 enhanced the intracellular transport of schisandrin B and deoxyschisandrin but decreased that of gomisin C, resulting in alteration of cell-killing bioactivity. The Second Law of Thermodynamics allows this type of unidirectional transport. Conclusively, SC-2 alters the transport and cell killing capability by a “Catcher-Pitcher Unidirectional Transport Mechanism”.

## Introduction

The primary function of polysaccharides is supposed only to assist tissue hydration and increase tissue resilience [1], [2]. Pharmaceutically, polysaccharides exhibit a diversity of uses including the drug transport improver [3], sustaining medicine transport [4], serving as an anchorage site for drug delivery liposomes [5], and enhancing the water solubility of carotenoids [6]. Hyaluronan, which was originally determined to act as intercellular glue, was recently found to be a very potent intracellular signaling agent associated with multiple drug resistance [2], immunity and oncology [7], [8].

*Schisandra chinensis* Turz Baill of the Magnoliaceae (*Wuweizi* in Chinese) (SC) is widely used as a valuable phytomedicine in China, Korea, and Japan to treat dysfunctional livers, lungs, hearts, and kidneys [9] and for chemical/viral hepatitis [10], [11]. Dibenzocyclooctadiene lignans isolated from SC (abbreviated as SCLs) include schizandrin, deoxyschisandrin (schisandrin A, SA), gomisins, schisandrol B, γ-schisandrin, wuweizisu B and C, and schisantherin C [12]. It is worth noting that the lignan compositional profile may depend on the separation technology [13].

Recently, SCLs like SA, Schisandrin B (SB) and gomisin C (GmC) have been well indicated to exhibit potent hepatoprotectives, anti-inflammatory, anticarcinogenics, antiviral, anti-HIV, immunomodulaters, and antioxidative properties [9], [11], [14]–[19]. By inhibiting P4503A4 activity, schisandrol A and gomisin A were shown to affect cellular drug metabolism and uptake. Biological studies indicated that an extract of SC seeds enhanced the hepatic glutathione (GSH) antioxidant/detoxification system and facilitated both processes in the livers, consequently considered to be a promising agent for improving phase I oxidative metabolism in CCl_4_-damaged livers [20]. Moreover, compound SB+sesamin preparation reveals a prominent *in vivo* hepatoprotective effect [21].

Recently, the bioactivity of soluble polysaccharide of *Schisandra* fruits was found to have potent immunomodulating properties, like improving the weight of immune organs and enhancing the phagocytic activity of peritoneal macrophages [22]. Yan et al. demonstrated a rather promising synergistic hepatoprotective effect of SCLs when co-administered with *Astragalus* polysaccharides [23]. Previously, we found the peptidoglycan (named SC-2) to be biologically inactive against the HepG2 cells (unpublished data). However, since SC-2 is water soluble in nature and decoction process has been always preferred for many Chinese Medicinal Preparations, we hypothesize that SC-2 with certain unknown mechanism might favor the therapeutic effect of SCLs. To verify this, the therapeutic effect of a serial model of SC-2, either used alone or in combination with individual SCLs, was extensively explored.

## Materials and Methods

### Isolation and purification of dibenzocyclooctadiene lignans

Desiccated sample SC fruits were purchased from Sun Ten Pharmaceutical Corp. (Taipei, Taiwan, ROC). Ten grams of desiccated fruits were extracted three times with 95% ethanol; each time 100 ml was extracted for 30 min in a sonication-assisted extractor. We have described the detailed methods in Text S1.

### High-performance liquid chromatographic (HPLC)/electrospray ionization (ESI)/tandem mass spectrometry (MS/MS) analyses

Separation of the dibenzocyclooctadiene lignans was conducted on a Luna C18(2) column (ℓ×i.d. = 2.00×150 mm, thickness = 3.0 µm) and a guard column (ℓ×id = 10×3 mm, Phenomenex Inc., Torrance, CA., U.S.A.) using an HPLC system consisting of a Finnigan Surveyor module separation system and a photodiode-array (PDA) detector (Thermo Electron Co., MA., U.S.A.). The next elution process and instrument setting was carried out according to La Torre et al. [24]. We have described the detailed methods in Text S1.

### Fourier transform infrared (FTIR) analyses of isolated lignans

The lignans SA, SB and GmC were separately desiccated under a vacuum at 40°C for 16 h, respectively mixed with KBr powder (IR grade) at a ratio lignan: KBr = 1∶ 100 (w/w) and fabricated into tablets. The tablet was scanned with Shimazdu 8400S FTIR 460 (Shimadzu, Tokyo, Japan) spectrophotometer against the KBr blank at 400∼4000 cm^−1^ and a resolution of 2 cm^−1^. Each sample was repeatedly scanned at least 10 times to assure the precision of the data. We have described the detailed methods in Text S1.

### Solvent extraction of crude polysaccharides from SC

The method for extraction of crude polysaccharides from SC (SC-CP) was carried out according to Ker et al. [25]. We have described the detailed methods in Text S1.

### Purification of crude polysaccharides from SC

Further isolation and purification of SC-CP were conducted with gel permeation chromatography (GPC) carried out according to Ker et al. [25] (be referred to Text S1). The yield of the purified product of the second fraction of SC-polysaccharide was 3.58%w/w (denoted as SC-2). We have described the detailed methods in Text S1 [26], [31].

### Characterization of the molecular weight and the molar extinction coefficient with high-performance size exclusion chromatography-tandem UV-visible and evaporative light scattering detection (HPSEC-UV-ELSD)

The HPSEC-UV-ELSD analysis was conducted to determine the molecular weight of SC-2. We have described the detailed methods in Text S1.

### X-ray powder diffraction (powder XRD) of SC-2

Desiccated purified SC-2 powder was macerated to fine, homogenous consistency and subjected to an X-Ray diffraction analyzer (X'Pert Pro MRD, PANalytical B. V., Almelo, The Netherlands). We have described the detailed methods in Text S1.

### FTIR analyses of purified SC-2 and pure lignans+SC-2

To measure the combined IR spectra, pure SC-2 alone was used as reference blank. The other combined formula were prepared by mixing each lignans with SC-2 at equimolar ratio, i.e. for SA+SC-2: 2 mL of SA solution (1.04 mg in 25 mL)+2 mL of SC-2 solution (1 mg mL^−1^). For SB+SC-2: 2 mL of SB (4 mg in 25 mL)+2 mL of SC-2 (4 mg mL^−1^); and for GmC+SC-2: 2 mL of GmC solution (5.2 mg in 25 mL)+2 mL of SC-2 solution (4 mg mL^−1^) were used. We have described the detailed methods in Text S1.

### Monosaccharide composition of SC-2

The method for analyzing the monosaccharide composition was based on previous work [25], [26]. We have described the detailed methods in Text S1 [25], [35].

### Amino acid composition in the protein moiety of SC-2

The method for analyzing the amino acid composition was according to previous work of Ker et al. [25]. We have described the detailed methods in Text S1 [25].

### Source and cell line

The human hepatocellular carcinoma cell line, HepG2 (BCRC 60380), was obtained from the Bioresource Collection and Research Center (BCRC, Food Industry Research and Development Institute, Hsin-Chu City, Taiwan). The detailed methods for cultivation and stocking are described in Text S1.

### Cell culture and cell viability assay

Cultivation of HepG2 cells and the MTT assay were performed as previously reported [27]. We have described the detailed methods in Text S1.

### Determination of the uptake rate of free SB, GmC, and deoxyschisandrin in the absence and presence of SC-2 by HepG2 cells

We have described the detailed methods in Text S1.

### Determination of the intracellular disposition of SC-2 in HepG2 cells

#### Fluorescein isothiocyanate (FITC) labeling of SC-2

Methods of Kanebo et al. [28] and Tanaka et al. [29] were followed with slight modification. We have described the detailed methods in Text S1.

#### Intracellular disposition of FITC-labeled SC-2

HepG2 cells (1×10^5^ cells/mL) were seeded onto a 3.5 cm dish containing 2 mL of DMEM medium. After incubated for 24 h at 37°C, FITC-SC-2 at 0.01, 0.1, 1.0, 10.0, and 25 µg mL^−1^ were added and incubated to investigate the dose- and time–dependent effects on the disposition of SC-2. We have described the detailed method in Text S1 [28], [29].

### Terminal deoxynucleotidyl transferase-mediated dUTP nick end-labeling (TUNEL) assay

A TUNEL assay using the Fluorescein Apoptosis Detection Kits (Roche Applied Science, Indianapolis, IN, USA) was carried out according to the manufacturer's instructions by Borisov et al. [30]. We described the detailed methods in Text S1 [31].

### Statistical analysis

Data obtained in the same group were analyzed by an analysis of variance (ANOVA) and Student's *t*-test with computer statistical software SPSS 10.0 (SPSS, Chicago, IL, USA). Statistical Analysis System (2000) software was used to analyze the variances, and Duncan's multiple-range test was used to test the significance of difference between paired means. The significance of the difference was judged by a confidence level of *p*<0.05.

## Results

### HPLC and ESI/MS/MS analysis of GmC Deoxyschisandrin (SA) and SB

**Figure 1:**
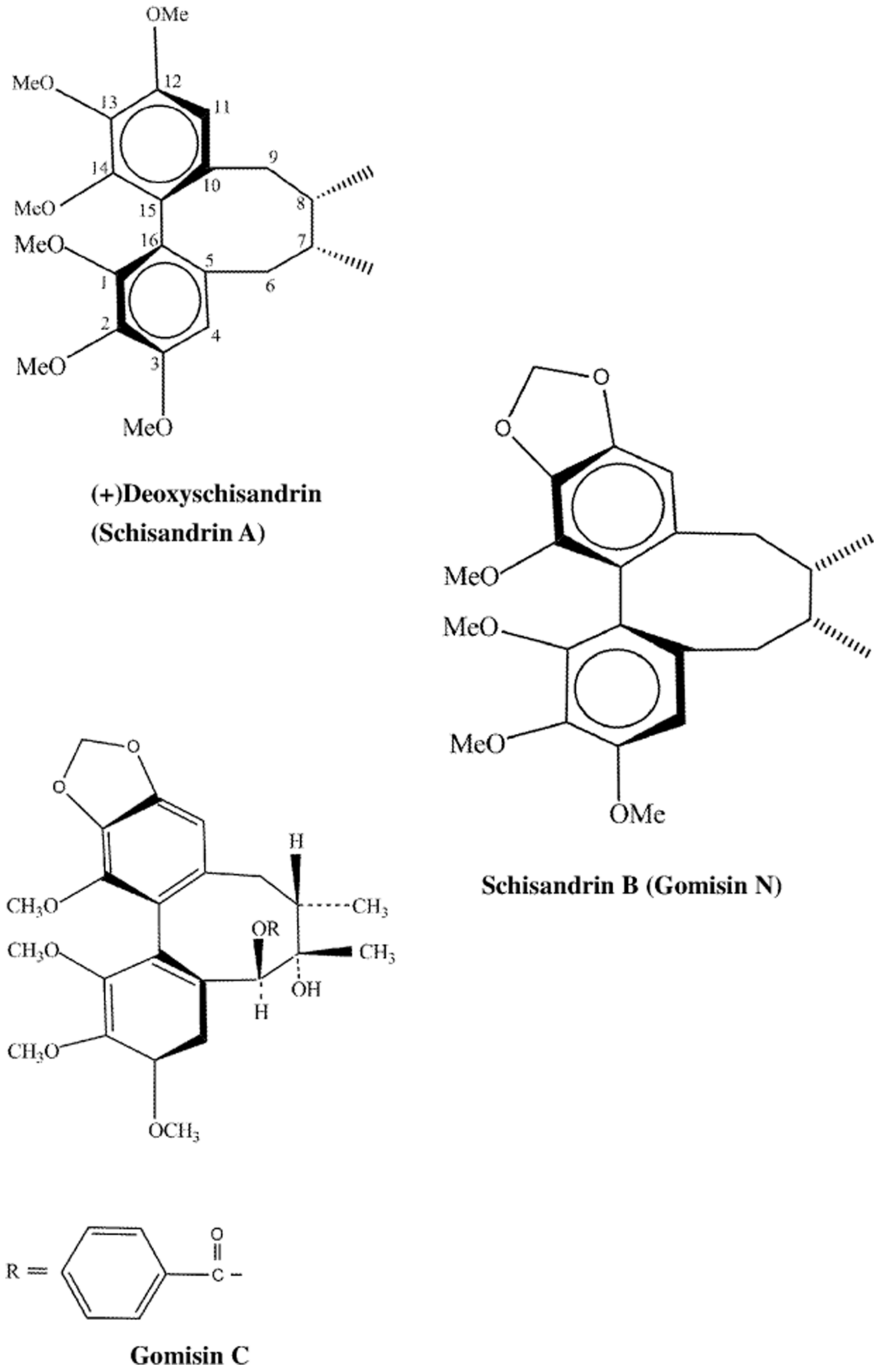
Chemical structures of liganas isolated from *S. chinensis* fruits. Gomisin C, deoxyschisandrin and schisandrin B isolated from the *S. chinensis* fruits. Structures of (+)deoxyschisandrin and (−)schisandrin B are depicted from Gnabre et al. (2010) [27]. Structure of gomisin C is depicted from Wang et al, (1994) [36].

**Figure 2:**
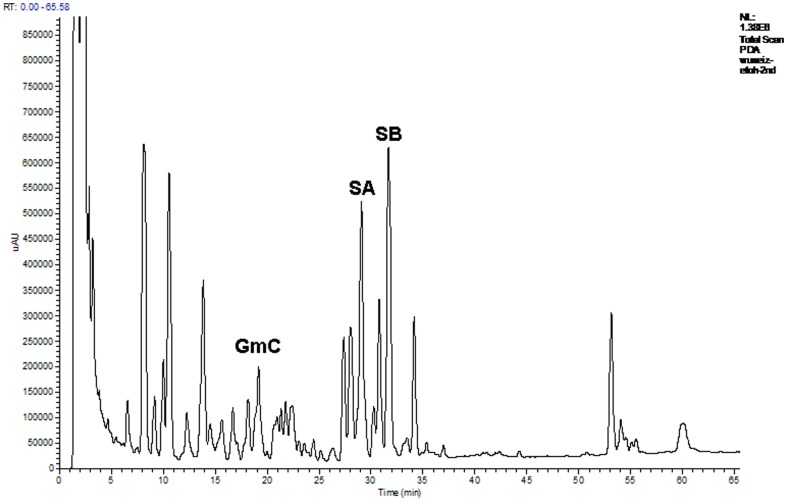
Spectral characterization of the peptidoglycan SC-2 isolated a from *S. chinensis* fruits. The absorption at 490(A). Purity identification of SC-2 obtained from the fractions 33–39 obtained by Sephadex G-100 column by high-performance size exclusion chromatography (HPSEC) with a PolySep GFC P-4000 column (300×7.8 mm) with water as eluant at a flow rate of 0.8 ml/min (B). The FTIR spectrum of purified SC-2. C (C): And the X-ray powder diffraction pattern of SC-2 (D).

The retention times of GmC, SA and SB (Fig. 1) in HPLC were 19.17, 29.07 and 31.69 min (Fig. 2) and their molecular weights were 536.6, 416.5 and 400.5, respectively (Fig. S1).

### Characterization of SC-2

**Figure 3:**
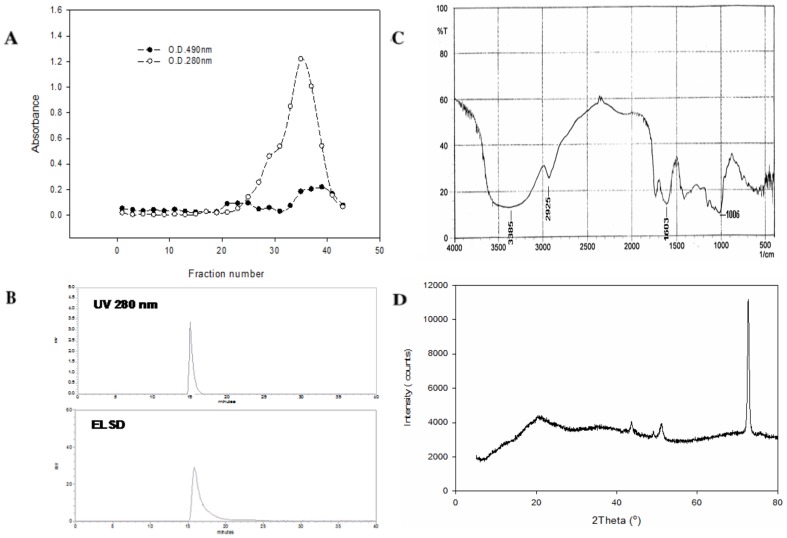
HPLC chromatographic analysis of the ethanolic extract of *S. chinensis* fruits. The retention times for the dibenzocyclooctadiene lignans were: gomisin C (GmC), 19.17 min; deoxyschisandrin (SA), 29.07 min; schisandrin B (SB), 31.69 min; gomisin O, 8.13 min; schizandrol B, 10.56 min; and gomisin R, 13.85 min, respectively.

The purified soluble polysaccharide, named hereafter SC-2, showed an overall yield 3.58% (Fig. 3A, Table 1). SC-2 exhibited a MW 841 kDa (Fig. 3B) and molar extinctions 1.0260×10^7^ M^−1^ and 1.766×10^7^ M^−1^ at 280 nm and 490 nm, respectively (Fig. 3A). It contained 28.20 wt% protein and 68.97 wt% carbohydrate, yielding a ratio protein/carbohydrate = 0.4089 (Table 1). The sugar portion of SC-2 contained 10 monosaccharide species (Table 1). Their contents (in mol%) were fucose (28.64), rhamnose (14.64), arabinose (13.74), xylose (13.32), glucose (11.12), allose (8.34), ribose (4.61), talose (2.13), mannose (1.84) and myoinsitol (1.29) respectively, but galactose was completely absent (Table 1). SC-2 comprised 15 kinds of amino acids. The major ten were (in %w/w): valine (10.34), leucine (15.80), isoleucine (14.29), methionine (1.98), proline (0.09)+hydroxyproline (1.96) ( = 2.05), phenylalanine (21.54)+tyrosine (2.94) ( = 24.48), cysteine (8.63), and histidine (17.15). The total essential amino acids amounted to 80.3%w/w (Table 1). The FTIR (KBr) ν_max_ (cm^−1^): spectrum showed many characteristic absorption bands, such as 3384.91 (ν_O-H_, s, broad, polyhydroxyl hydrogen bonding), 3144.07, 3087.17 (aromatic ν_C-H_, s), 2857.64 (ν_C-H_, CH_3_, m), 1734.17 (ν_C = O_, m), 1647.02 (ν_C = O_, amide, s), 1602.9, 1457.27 (δ_N-H_, amide, s), 1339.61 (ν_C-O_, amide, m), 1151.54 (ν_C-O_, alcohol, s), 1100∼1010 (ν_C-O_, β-pyranoside, s), 1075.35 (ν_C-O_, ester, s), 1006.88 (ν_C-O_, ether, s), 952.87, 854.49 (δ_C-H_, alkene, s), 768.66, 761.78 (δ_C-O_, β-glycosidic linkage, w) and 680–760 (δ_C-H_, aromatic, s)(Fig. 3C).

**Table 1:** The compositional analysis of the glycoprotein SC-2 purified from *S. chinensis* fruits.

Overall yield of purified SC-2, %w/w	3.58
Mean molecular weight, kDa	841
**Proximate analysis (%w/w/)**	
Total carbohydrate content	68.97
Crude protein content	28.20

Monosaccharide and amino acid composition of SC-2			
monosaccharide	Content (mol%)	Amino acid	Content (%w/w)
Rhamnose	14.64	Alanine	0.18
Fucose	28.97	Glycine	0.40
Ribose	4.61	Valine	10.34
Arabinose	13.74	Leucine	15.80
Xylose	13.32	Isoleucine	14.29
Allose	8.34	Proline	0.09
Talose	2.13	Methionine	1.98
Mannose	1.84	Glutamic acid	0.38
Galactose	n.d	Hydroxyproline	1.96
Glucose	11.12	Phenylalanine	21.54
Myo-inositol	1.29	Cysteine	2.60
		Lysine	8.63
		Tyrosine	2.94
		Aspartic acid	1.72
		Histidine	17.15

n.d.: not detected.

The powder XRD pattern revealed three 2θ peaks, i.e. 2θ_1_ = 42.2160° with an intensity of 3644; 2θ_2_ = 51.1398° with an intensity of 3938; and 2θ_3_ = 72.66393° with intensity of 11190, giving the specific diffraction angles at θ_1_ = 21.1080°, θ_2_ = 25.5699°, and θ_3_ = 36.3319°, respectively (Fig. 3D).

### FTIR and UV-Vis- ESI-MS- ESI-MS-MS characterization of the isolated lignans

For free SA: UV λ_max_ (nm): 234, 254 (sh); ESI(+)-MS (m/z): 417 [M+H]^+^ (Fig. S1).

**Figure 4:**
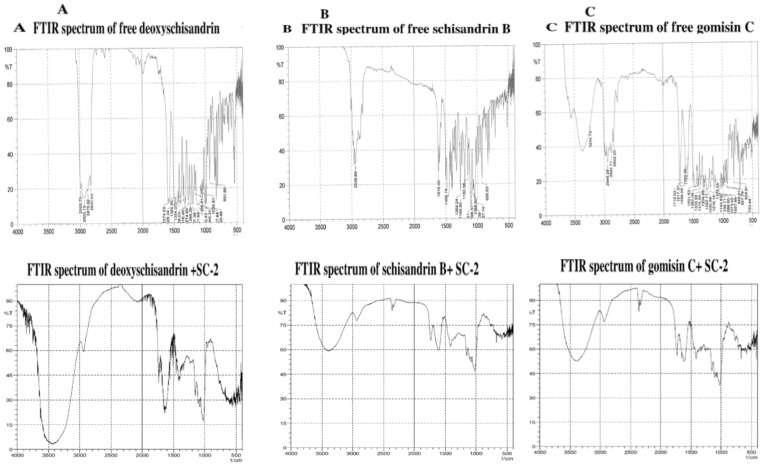
FTIR spectra of the purified free lignans and the lignans+SC-2. Purified free deoxyschisandrin (upper panel) and deoxyschsandrin+SC-2 (lower panel) (A). Purified free schisandrin B (upper panel) and schisandrin B+SC-2 (lower panel) (B). And purified free gomisin C (upper panel) and gomosin C+SC-2 (lower panel) (C). In measuring of the combined IR spectra, equimolar amount of each was used: for deoxyschisandrin+SC-2: 2 mL of deoxyschisandrin solution (1.04 mg/25 mL)+2 mL of SC-2 solution (1 mg/mL). For schisandrin B+SC-2: 2 mL of schisandrin B (4 mg/25 mL)+2 mL of SC-2 (4 mg/mL). And for gomisin C+SC-2: 2 mL of gomisin solution (5.2 mg/25 mL)+2 mL of SC-2 solution (4 mg/mL) were used. The mixture was respectively mixed thoroughly with KBr (IR grade) (in 1∶100 w/w), dried at 40°C under vacuum for 16 h, fabricated into KBr tablets and subjected to FTIR scanning using Shimadzu FTIR 460 (Shimadzu, Tokyo, Japan). Each sample was repeatedly scanned for at least 10 times to assure the precision of the data.

IR (KBr) ν_max_ (cm^−1^): 2990.73, 2925.15, 2876.92, 2830.63 (ν_C-H_, CH_3_, s), 1574.93, 1428.34, 1385.90, 1354.07, 1328.03, 1316.46, 1274.99, 1269.20, 1201.69 (ν_C-O_, phenolic, s), 1166.01, 1100.43, 1049.31, 1035.81, 1007.84 (ν_C-O_, etheric, s), 990.48, 931.65 (δ_C-H_, s, 2 peaks) (Fig. 4A, upper panel).

For free SB: UV λ_max_ (nm): 237, 262 (sh); ESI(+)-MS (m/z): 401 [M+H]^+^ (Fig. S1).

IR (KBr) ν_max_ (cm^−1^): 2970.00, 2938.38, 2830.00 (ν_C-H_, CH_3_, s), 1616.4 (aromatic ν_C-H_, s), 1498.74, 1268.24 (phenolic ν_C-O_, s), 1198.80, 1150.58, 1106.21, 1096.57, 1068.60, 1047.38, 1037.74 (etheric ν_C-O_, s), 926.83, 937.22 (δ_C-H_, s, 2 peaks) (Fig. 4B, upper panel).

For free GmC: UV λ_max_ (nm): 236, 258 (sh); ESI(+)-MS (m/z): 537 [M+H]^+^ (Fig. S1). IR (KBr) ν_max_ (cm^−1^): 3600.00 (ν_O-H_, alcohol s), 3234.73 (ν_O-H_, phenolic s, m broad H-bonding), 2948.29, 2882.71, 2842.20 (ν_C-H_, s), 1718.63 (ν_C = O_, s), 1617.37, 1598.08, 1582.65, 1501.63 (ν_C = C_, aromatic, s), 1382.04, 1333.82 (ν_O-H_, phenolic, s, 2 peaks), 1282.71, 1250.88, 1216.16, 1178.55, 1143.83, 1109.11, 1098.50, 1073.42, 1057.99 (ν_C-O_, etheric, s), 949.01, 927.79 (δ_C-H_, alkene, s), 818.81 (δ_C-H_, polysubstituted aromatic, s) and 720.44 (δ_C-H_, C6-monosubstituted aromatic, s) (Fig. 4C, upper panel).

### FTIR spectra of the combined lignans plus SC-2

Surprisingly, we found the FTIR spectra of the combined lignans+SC-2 (Fig. 4) to be rather similar to that of SC-2 (Fig. 2C) alone.

For SA+SC-2, IR (KBr) ν_max_ (cm^−1^): 3384.92 (ν_O-H_, s, broad, polyhydroxyl hydrogen bonding), 2925.15 (ν_C-H_, CH_3_O-, m), 1750 (ν_C = O_, esteric, s), 1608–1650, (ν_C = O_, amide, m) 1420 1400 (ν_C-N_, amide, m), 1360 (ν_C-O_, amide, m), 1200 (ν_C-O_, phenolic, s), 1150, 1105 (ν_C-O_, alcoholic, s), 1105, 1023 (ν_C-O_, β-pyranoside, s). 768.34, 761.28 (δ_C-O_, β-glycosidic linkage, w), 760–690 (δ_C-H_, aromatic, disappeared or masked by SC-2) (Fig. 4A, lower panel).

For SB+SC-2, IR (KBr) ν_max_ (cm^−1^): 3400.30 (ν_O-H_, s, broad, polyhydroxyl hydrogen bonding), 2938.65 (ν_C-H_, CH_3_O-, m), 2385.30, 2379.24 (ν_C-H_, -CH = N-, m), 1740.33 (ν_C = O_, s), 1615.80 (ν_C-H_, conjugated alkene, s), 1400.02 (ν_C-N_, amide, s), 1160.50, 1030.34 (ν_C-O_, ether, s), 760–690 (δ_C-H_, aromatic, disappeared or masked by SC-2) (Fig. 4B, lower panel).

For GmC+SC-2, IR (KBr) ν_max_ (cm^−1^): 3485.26 (ν_O-H_, s, broad, polyhydroxyl hydrogen bonding), 2948.59 (ν_C-H_, CH_3_O-, m), 2480.00, 2300.30 (ν_C-H_, -CH = N-, m), 1724.20 (ν_C = O_, esteric, s), 1612.20 (ν_C = O_, amide, s), 1425.00 (ν_C-N_, amide, s), 1160.11, (ν_C-O_, alcoholic, s), 1026.22 (ν_C-O_, etheric, s; ν_C-O_, β-pyranoside, s), 760–690 (δ_C-H_, aromatic, disappeared or masked by SC-2) (Fig. 4C, lower panel).

### Effect of SC-2 on the cell viability of HepG2 cells

**Figure 5:**
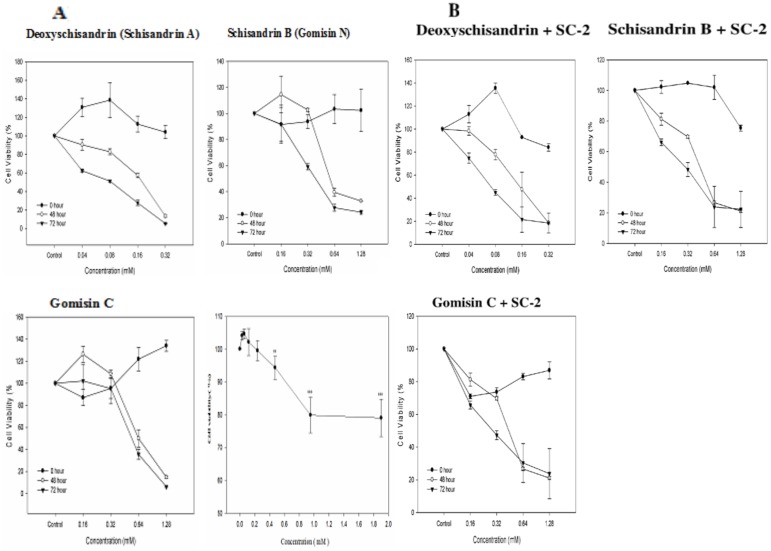
Effect of free lignans and the peptidylglycan SC-2 on HepG2 cell viability. The cell viability affected by the free lignans and SC-2 (A). And the cell viability affected by the combined therapy of ligna+SC-2 (B). For free SC-2 in figure 5A, the cells were cultured in 10% FBS medium and treated with SC-2 at dosages 0.0297 mM, 0.0595, 0.1189, 0.2378, 0.4756, 0.9512, and 1.9024 mM, respectively for 48 h. The percent cell viability was calculated by comparing with the control (arbitrarily set as 100%) Values are expressed as mean±S.D. of triplicate independent experiments (**p<0.01 and ***p<0.001 *vs* control group).

In medium containing 10% FBS, when treated with SC-2 at dosages 0.0297, 0.0595, 0.1189, 0.2378, 0.4756, 0.9512, and 1.9024 mM, respectively, the cell viability was seen still retaining at a level >80%, indicating the totally nontoxic behavior of SC-2 to HepG2 cells. At higher doses (0.9512∼1.9024 mM), a slightly declined cell viability occurred, suggesting a masking or plugging effect of SC-2 on the cell membrane (Fig. 5D).

### Pharmacokinetic behavior of lignans in HepG2 cells

The uptake rates of free *Shisandra* lignans by HepG2 cells greatly differed from those combined with SC-2. For SB and SA, the uptake rates were apparently elevated by presence of SC-2. On the contrary, GmC showed a lower uptake rate (Table 2).

**Table 2:** Enhanced lignan uptake rate mediated by glycoprotein SC-2.

Incubation time, (min)	Schisandrin B		Schisandrin B+SC-2^a^	
	Uptake (µM)	Decay rate constant, k_1_(min^−1^)	Uptake (µM)	Decay rate constant, k_2_ (L•mmol^−1^ min^−1^)
0	47^b^	-	46^b^	-
5	54±2	0.0140^b^	58±2	2.016×10^−5^ b
15	68±3	0.0140	82±2^**^	2.016×10^−5^
30	76±3	0.0053	86±4^*^	2.269×10^−6^
60	82±3	0.0020	91±5	1.429×10^−6^
90^b^	86^b^	0.0013^b^	93^b^	5.865×10^−7^ b
120^b^	82^b^	−00013^b^	89^b^	−1.092×10^−6^ b

Incubation time, (min)	Gomisin C		Gomisin C+SC-2	
	Uptake (µM)	Decay rate constant, k_1_ (min^−1^)	Uptake (µM)	Decay rate constant, k_2_ (L•mmol^−1^ min^−1^)
0	24^b^	-	10^b^	-
5	25±1	0.0020^b^	13±1**	5.046×10^−6^ b
15	27±3	0.0020	19±4*	5.046×10^−6^
30	27±1	0.0000	19±3 *	0.0000
60	23±3	−0.1333	15±1	−1.092×10^−6^

Incubation time, (min)	Deoxyschisandrin		Deoxyschisandrin+SC-2	
	Uptake (µM)	Decay rate constant, k_1_ (min^−1^)	Uptake (µM)	Decay rate constant, k_2_ (L•mmol^−1^ min^−1^)
0	43^b^	-	55^b^	-
5	48±2	0.0010^b^	62±0.4^**^	1.264×10^−5^ b
15	58±4	0.0010	77±5^**^	1.264×10^−5^
30	63±6	0.0033	74±5	1.976×10^−6^
60	59±2	−0.0013	70±2	−1.092×10^−6^

a Dose of SC-2 (MW: 841 kDa): 1 mg/mL ( = 1.1891×10^−3^ mM) (^*^ *p*<0.05; ^**^ *p*<0.01).

b Values obtained by extrapolation.

Worth noting, free SA uniquely revealed a relatively delayed uptake rate that was totally not seen for the others (Table 2). In contrast, the uptake process was relatively shorter for both GmC and SA. Their peak points in uptake rates reached at or around 30 min (Table 2). Intracellular GmC was rapidly consumed up at 30 min for GmC and at 60 min for SA. The intracellular decay rate coefficient was ∼1.092×10^−6^ L•mmol^−1^ min^−1^ for both GmC and SA (Table 2). A relatively longer uptake time was required by SB, which remained at 1.429×10^−6^ L•mmol^−1^ min^−1^ even at 60 min (Table 2). The total amounts delivered from the extracellular to the intracellular compartments in the presence of SC-2 were (in decreasing order) SB>SA>GmC, corresponding to 91±5 µM (at 60 min)>77±5 µM (at 15 min)>19±3 µM (at 30 min), respectively. For comparison, the respective order of the free lignans was: 82±3, 63±6 and 27±1 µM. Interestingly, in the presence of SC-2 the uptake of GmC was significantly retarded (Table 2).

### Pharmacodynamic behavior of lignans in HepG2 cells

Behaviors of HepG2 cells responding to these three lignans varied greatly depending on the dose, time of incubation, and the presence or absence of SC-2. At 48 h, free SB alone showed activated cell proliferation within doses of <0.16 mM (Fig. 5A). In the presence of SC-2, this activation disappeared (Fig. 5B). A similar phenomenon was seen for GmC (Fig. 5A & 5B), but not for SA (Fig. 5A & 5B). The IC_50_ values at 48 h were 0.55 mM, 0.64 mM and 0.19 mM without SC-2, while they significantly improved to 0.41. 0.51, and 0.15 mM, for SB, GmC and SA in the presence of SC-2, respectively (Table 3). Comparing to data at 48 h, the IC_50_ values at 72 h had further improved to 0.47 mM, 0.58 mM and 0.08 mM for the free lignans SB, GmC and SA (Table 3, Fig. 5A), and to 0.32 mM, 0.29 mM and 0.08 mM for SC-2+lignans, respectively (Table 3, Fig. 5B).

**Table 3:** The cytotoxicity and HepG2 cell killing-capability of dibenzocyclooctadiene lignans in the presence and absence of its coexisting glycoproteinSC-2^a^.

	Incubation time, h			
Treatment	48		72	
	IC_50_, mM	Killing capability, cells/mM^b^	IC_50_, mM	Killing capability, cells/mM^b^
Schisandrin B	0.55±0.06	1.11×10^5^ cells/mM (within 0.16–1.28 mM)	0.47±0.04	1.52×10^5^ cells/mM (at <0.64 mM)
Schisandrin B+SC-2	0.41±0.03	1.82×10^5^ cells/mM (at <0.64 mM)	0.30±0.14	1.76×10^5^ cells/mM (at <0.64 mM)
Gomisin C	0.64±0.01	1.46×10^5^ cells/mM (at >0.16 mM)	0.58±0.02	1.42×10^5^ cells/mM (within 0.32–1.28 mM)
		1.73×10^5^ cells/mM		
Gomisin C+SC-2	0.51±0.02	(at <0.64 mM; average of two phases)	0.29±0.04	3.94×10^5^ cells/mM (at ≤1.28 mM)
Deoxyschisandrin	0.20±0.00	3.94×10^5^ cells/mM(at <0.32 mM)	0.10±0.00	4.59×10^5^ cells/mM (at <0.32 mM)
Deoxyschisandrin+SC-2	0.15±0.04	4.29×10^5^ cells/mM(at <0.32 mM)	0.07±0.01	7.50×10^5^ cells/mM (at <0.32 mM)

a Dose of SC-2 (MW: 841 kDa): 1 mg/mL ( = 1.1891×10^−3^ mM) (^*^ *p*<0.05; ^**^ *p*<0.01).

b Killing capability was measured within the linearity range of viability-dose in Fig. 5A and 5B.

At 48 h, the respective killing capabilities were found to be 2.93×10^5^ cells/mM, 1.46×10^5^ cells/mM and 3.94×10^5^ cells/mM when used alone. The combined use with SC-2 obviously altered the cytotoxic effects to 1.71×10^5^ cells/mM, 1.73×10^5^ cells/mM and 4.29×10^5^ cells/mM, respectively for SB, and GmC and SA (Table 3). However at 72 h, the killing capabilities of free SB and free GmC were only comparable to those at 48 h. Conversely, SC-2 astonishingly significantly enhanced the cytotoxicity of GmC and SA to 3.94×10^5^ cells/mM and 7.50×10^5^ cells/mM, respectively (Table 3, Fig. 5A & 5B).

### The SC-2 peptidoglycan was not transportable through the HepG2 cell membrane

**Figure 6:**
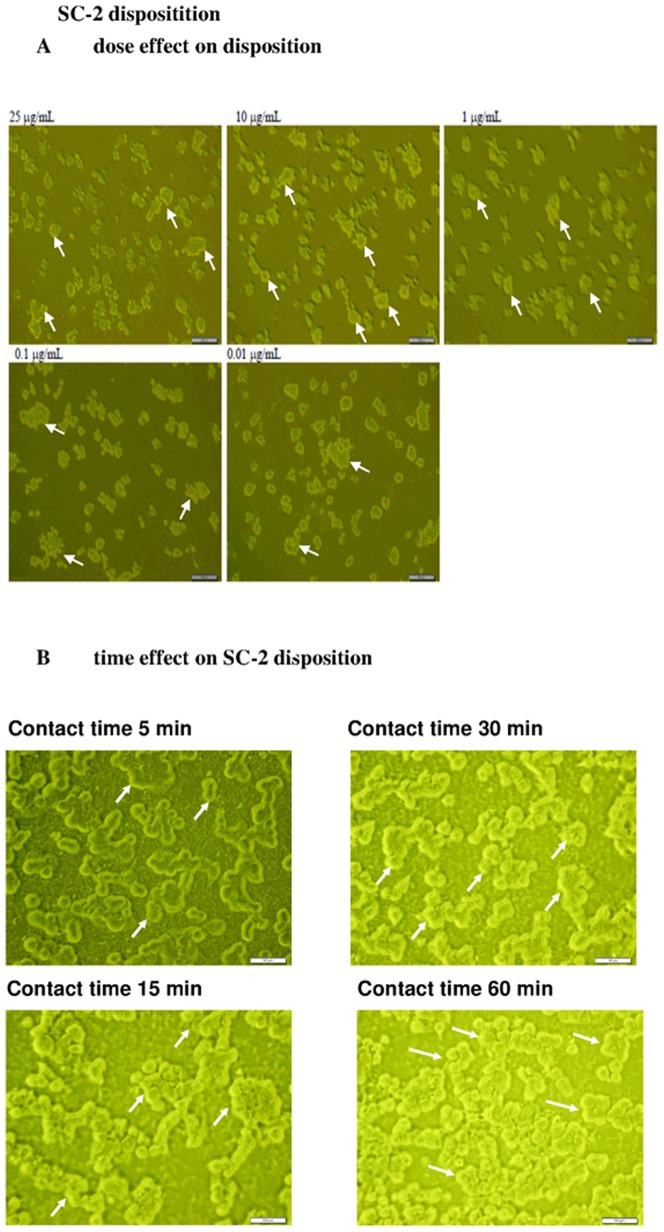
Fluorescent labeling technique to investigate the intracellular deposition of SC-2 into the HepG2 cells (×400). The dose effect (A), and the time effect (B). SC-2 was covalently labeled in equimolar ratio with FITC to form FITC-SC-2. In experiment A: Hep G2 cells at 1×10^5^ cells/mL were seeded onto 3.5 cm plate containing 2 mL of DMEM and incubated for 24 h. FITC-SC-2 at 0.01, 0.1, 1.0, 10, and 25 µg/mL was added, and the incubation was continued for 30 min. In experiment B: Hep G2 cells at 1×10^5^ cells/mL were seeded onto 3.5 cm plate containing 2 mL of DMEM and incubated for 24 h. FITC-SC-2 (10 µg/mL) was added, and the incubation was continued and sampled at the hour as indicated. As seen, in both experiments the FITC-SC-2 probes remained exclusively onto the outer membrane. Blank arrows indicate the non-fluorescent intracellular compartment.

SC-2 was totally nontoxic when used alone at a wide range of dosages (Fig. 5A), implying that free SC-2 was not mobilized into HepG2 cells. The florescent technology revealed the FITC-SC-2 molecules exclusively remained on the outer membrane of HepG2 cells even after 30 min of contact. No apparent difference was seen from the dose effect (Fig. 6A). However, the time-effect showed distinct higher accumulation of SC-2 on cell membrane (Fig. 6B).

### TUNEL Assay

**Figure 7:**
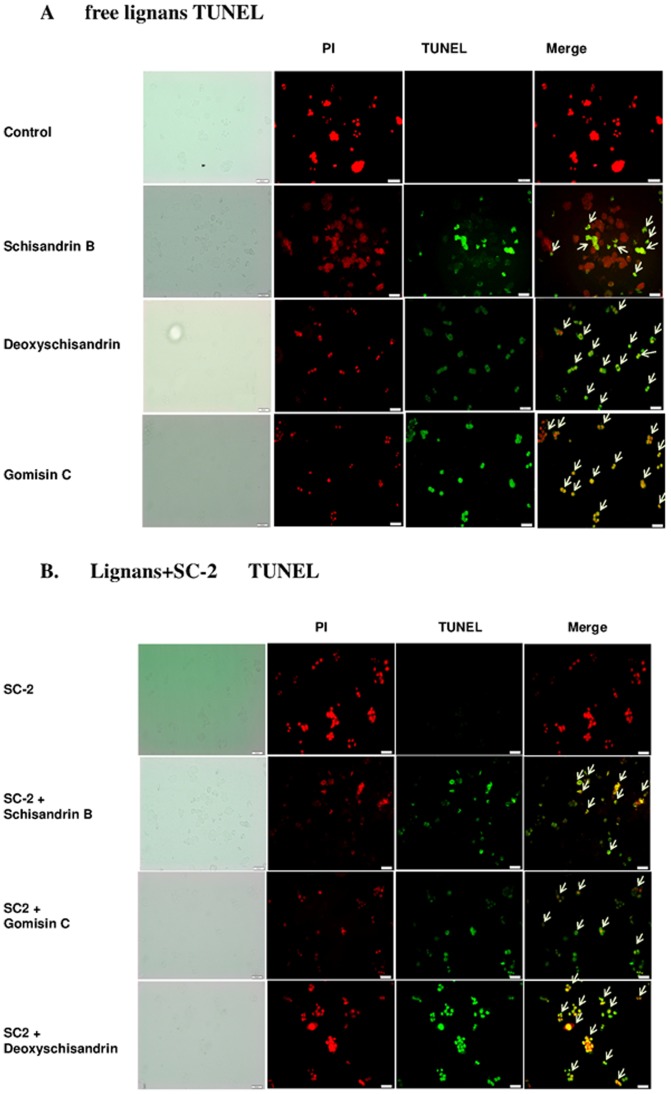
TUNEL assay for HepG2 cells. Cells treated with free lignans (A), and lignan plus SC-2 (B). Cells were induced for 24 h, then PI staining and TUNEL assay were carried out. Results were examined under a fluorescence microscope (×400).

Free lignans were shown to be very effective in inducing apoptosis of HepG2 cells. DNA fragmentation was clearly perceivable by the TUNEL assay (Fig. 7A). In the presence of SC-2, the number of apoptotic cells was seen to have significantly increased (Fig. 7B).

### Thermodynamic consideration of the transport process

**Figure 8:**
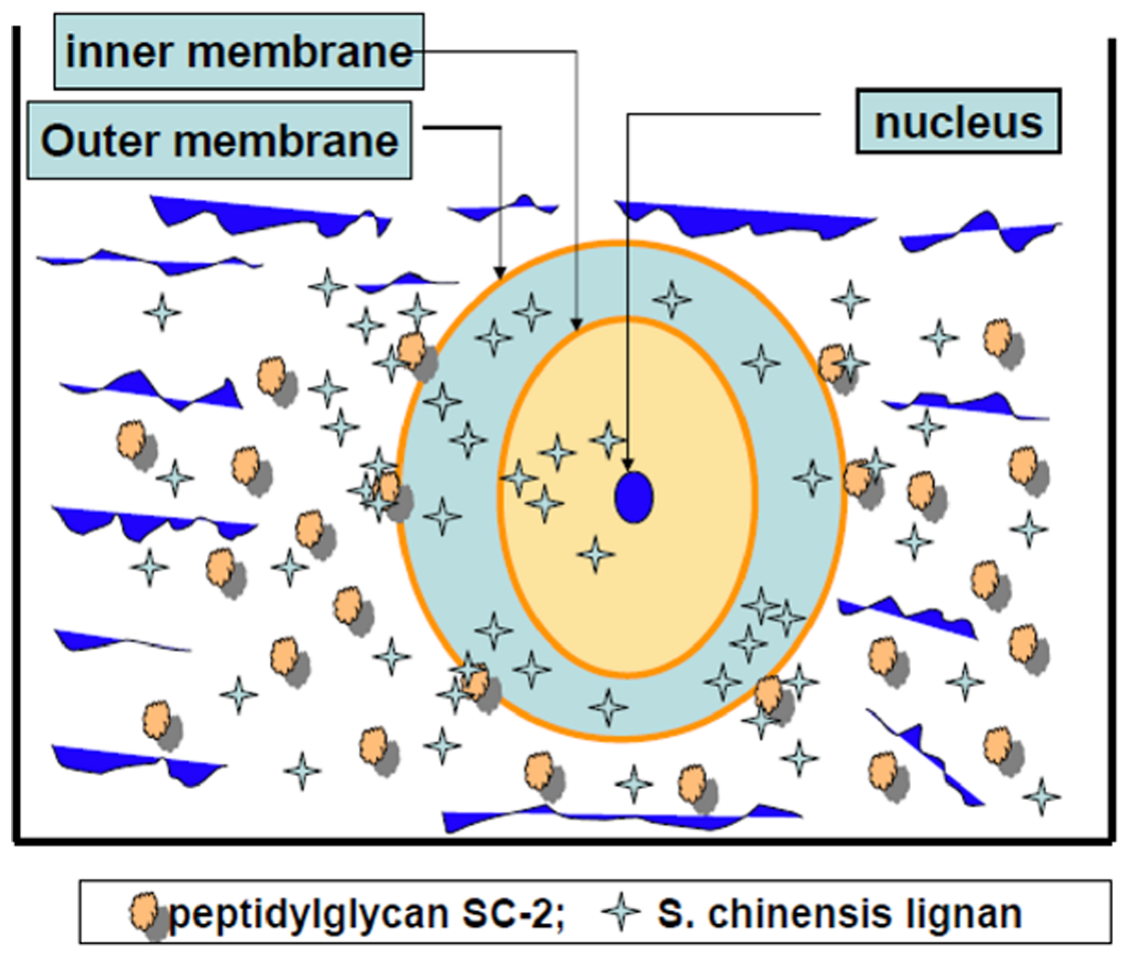
Diagrammatic model showing the transport of *S. chinensis* lignans through the HepG2 cell membrane in the presence and absence of peptidylglycan SC-2. The conformation of SC-2 was specifically altered when submerged onto the outer membrane of target cells, concomitantly, the free energy change declined to ΔG<0. The membrane-bound SC-2 specifically accumulated the lignans and pumped them into the intramembrane space. The cytosolic lignan concentration was thus rapidly raised to a higher level than the original extracellular concentration. Supposedly, Gomisin C bearing an OH-group at position 7 (Fig. 1) could be more tightly arrested by SC-2.

**Figure 9:**
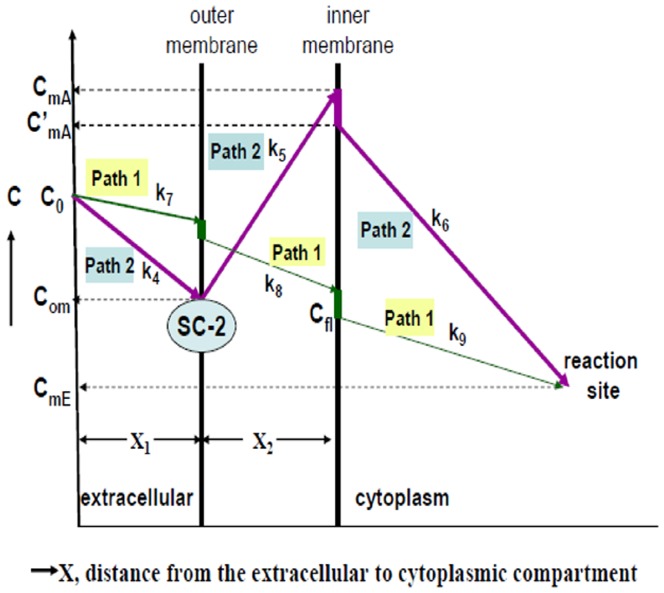
Two different transport mechanisms with detailed concentration changes along the paths. In path 1, the initial bulk fluid concentration of lignans (initial concentration C_0_) was passively transported a distance of X_1_ through the bulk fluid (reaction constant k_7_) and the cell membrane (thickness X_2_, reaction constants k_8_) to reach the inner membrane where due the membrane barrier the concentration dropped sharply to the effective innermembraneous concentration C_fl_, which was then moved into the cytoplasmic compartment and degraded (reaction constant k_9_) to C_mE_ at the reaction site of intracellular compartment (Fig. 9). Path 2 is the SC-2-assisted transport in which lignans in the bulk fluid (concentration, C_0_) were rapidly taken up by SC-2 already conjugated with the outer membrane (through a distance X_1_, reaction constant k_4_), where the outer membrane concentration rapidly dropped to C_om_. Due to the “actively” pumping effect of SC-2, the intramembrane lignan concentration was rapidly raised to C_mA_ (through a distance of membrane thickness X_2_, reaction constant k_5_), which, on moving along the inner membrane barrier, abruptly dropped down to C′_mA_ and simultaneously transferred into the cytoplamic compartment and soon degraded to attain the final concentration C_mE_ at the reaction site (reaction constant k_6_).

To give a clear image of the role of SC-2 in the transport process for lignans, we proposed the diagrammatic model shown as Figure 8, which demonstrates the transport model of lignans through HepG2 cell membrane in the presence and absence of SC-2. It was assumed that the conformation of SC-2 was specifically altered when submerged onto the outer membrane of target cells, concomitantly, the free energy change declined to ΔG<0. The membrane-bound SC-2 specifically accumulated the lignans and pumped them into the intramembraneous space. The cytosolic lignan concentration was thus rapidly raised to a higher level than the original extracellular concentration. Supposedly, GmC bearing an OH-group at position 7 (Fig. 1) could be more tightly arrested by SC-2. To quantify the magnitude of free energy changes, we defined two paths that transported lignans (Fig. 9), i.e. the path 1, in the absence of SC-2; and the path 2, in the presence of SC-2 In reality, path 1 is the common passive transport of lignans in the absence of SC-2. In path 1, the initial bulk fluid concentration of lignans (initial concentration C_0_) was passively transported a distance of X_1_ through the bulk fluid (reaction constant k_7_) and the cell membrane (thickness X_2_, reaction constants k_8_) to reach the inner membrane where due the membrane barrier the concentration dropped sharply to the effective innermembraneous concentration C_fl_, which was then moved into the cytoplasmic compartment and degraded (reaction constant k_9_) to C_mE_ at the reaction site of intracellular compartment (Fig. 9). Path 2 is the SC-2-assisted transport in which lignans in the bulk fluid (concentration, C_0_) were rapidly taken up by SC-2 already conjugated with the outer membrane (through a distance X_1_, reaction constant k_4_), where the outer membrane concentration rapidly dropped to C_om_. Due to the “actively” pumping effect of SC-2, the intramembraneous lignan concentration was rapidly raised to C_mA_ (through a distance of membrane thickness X_2_, reaction constant k_5_), which, on moving along the inner membrane barrier, abruptly dropped down to C′_mA_ and simultaneously transferred into the cytoplasmic compartment and soon degraded to attain the final concentration C_mE_ at the reaction site (reaction constant k_6_).

The elucidation for thermodynamic mathematical model is shown in Text S1. From the initial total extracellular concentrations and the extracellular and the intracellular concentrations at the pseudoequilibrium state (Table 2), the estimated parameters were obtained. The peak concentration was the highest for SB followed by GmC and SA (Table 4). By following the model presented in Figure 8 and Figure 9, the magnitude of the stepwise free energy change for each transport step was calculated (Table 5, see Text S1), from which the overall free energy change exampled by the largest ΔG_3_ of SA (Table 2, Table 4) was achieved (Table 6). As can be expected, the other overall free energy changes would also remain at values of ΔG_overall_ = −∞ (Table 6).

**Table 4:** Estimation of the parameters at status of pseudo equilibrium.

Parameter	Initial totalextracellular concentration	Extracellular concentration (mM)	Intracellularconcentration, Ls_in_ (mM)
[SC-2]^a^	1 mg/mL (1.1891×10^−3^ mM)	0.0	0.0
[M_outer_]	[M_outer_]>>[SC-2], hence [M_outer_]	[M_outer_]>>[SC-2], hence [M_outer_]	-
	≈constant	≈constant	
[SC-2-M_outer_]	1.1891×10^−3^ mM	1.1891×10^−3^ mM	0.0
Ls^c^:	C_0_ = 0.1 mM	-	(peak concentration)^c^
Schisandrin B	C_0_ = 0.1 mM	C_0m_ = 0.007 mM	C_mA_′ = 0.093 mM;
Gomisin C	C_0_ = 0.1 mM	C_0m_ = 0.081 mM	C_mA_′ = 0.019 mM;
Deoxyschsandrin	C_0_ = 0.1 mM	C_0m_ = 0.085 mM	C_mA_′ = 0.015 mM;
			Assume C_mA_′
			≈C_mA_(Fig. 8)
[SC-2-M_outer_]-Ls	1.1891×10^−3^ mM	1.1891×10^−3^ mM	0.0
C_mE_ b ^,^ c:	-	-	-
Schisandrin B	-	-	0.005 mM
Gomisin C	-	-	0.005 mM
Deoxyschsandrin	-	-	0.005 mM

a SC-2: MW = 841 kDa. 1 mg/mL = 1.1891×10^−3^ mM.

b C_mE_: estimated from Fig. 9.

c Be referred to Text S1, Table 2 and Fig. 9.

**Table 5:** Magnitude of parameters related with the free energy changes during the transport of lignans in the absence or presence of SC-2.

Parameters	Values of change in free energy, kcal/mol			
	ΔG_0,1_	ΔG_0,2_	ΔG_0,3_	ΔG_0,4_
[SC-2]^a^	≈0.0	-	-	-
[M_outer_]^b^	Constant K	-	-	-
[SC-2-M_outer_], M	≈1.1891×10^−6^	≈1.1891×10^−6^	-	-
[Ls], M	-	9.988×10^−4^		
[SC-2-M_outer_]-Ls, M		≈1.1891×10^−6^	≈1.1891×10^−6^	
[Ls_in_]^c^, M	-	-	-	-
Schisandrin B	-	-	46×10^−6^	46×10^−6^
Gomisin C	-	-	10×10^−6^	10×10^−6^
Deoxyschisandrin	-	-	55×10^−6^	55×10^−6^
C_om_, M	-	-	-	-
Schisandrin B			C_0m_ = 7×10^−6^	
Gomisin C			C_0m_ = 81×10^−6^	
Deoxyschsandrin			C_0m_ = 85×10^−6^	
C_mA_′, M	-	-	-	
Schisandrin B			C_mA_′ = 93×10^−6^;	
Gomisin C			C_mA_′ = 19×10^−6^;	
Deoxyschsandrin			C_mA_′ = 15×10^−6^	
C_mE_ d, M	-	-	-	-
Schisandrin B				5×10^−6^
Gomisin C				5×10^−6^
Deoxyschsandrin				5×10^−6^

a SC-2: MW = 841 kDa. 1 mg/mL = 1.1891×10^−6^M.

b The concentration of SC-2 was very small compared to that of outer membrane, hence [M_outer_] was considered to be constant and designated K.

c Values obtained by extrapolation to zero time zero.

d C_mE_: estimated from Fig. 6.

**Table 6:** The overall free energy changes during the transport of *S. chinensislignans* from the extracellular into the intracellular compartment^a^.

Parameters				
	ΔG_0,1_	ΔG_0,2_	ΔG_0,3_	ΔG_0,4_
Free energy changes, KJ	−∝	−2.572	+0.059^c^	−∝
K_eq_, mol^−1^	K_eq′_ = K_eq_×K = +∝^b^	0.9980	−0.0189	+∝^d^
Equation applied	Eq. 3–Eq. 6	Eq. 7–Eq. 9	Eq. 11, Eq. 12	Eq. 13–Eq. 16
Overall free energy change = ΔG_0,1_+ΔG_0,2_+ΔG_0,3_+ΔG_0,4_ = −∝				

a Free energy changes = −RTℓnK_eq_(J). R = 8.314 JK^−1^ mol^−1^. T = 310 K.

b K_eq′_ is a pseudoequilibrium constant. K is the amount of outer membrane concentration defined in Table 5.

c Value of ΔG_3_ was exampled by the largest value (of deoxyschisandrin) among these three lignans (be referred to Table 2).

d The value K_eq_ in calculation of ΔG_0,4_ in reality is not an equilibrium constant because reversible reaction does not occur in the intracellular degradation process. The value was estimated by the difference between the initial and the final conditions (be referred to text).

## Discussion

The powder XRD (Fig. 3D) revealed SC-2 to be high pure microcrystalline lattice structures with characteristic intra-lattice dimensions of d_1_ = 2.139 Å, d_2_ = 1.786 Å, and d_3_ = 1.300 Å. Speculatively, the strongest diffraction of θ_3_ could be due to diffraction from the main plane with alpha helical units lining up to elicit an inter-unit distance of 1.300 Å. While the other two distances, d_1_ and d_2_, may have been due to diffractions at secondary minor lattice planes. The specific molar extinction coefficients, the characteristic ratio of proteins to carbohydrates ( = 0.4089) and the strong hydrogen bonding and amide absorption bands as well as the β-glycosidic linkage absorption bands at 768.66 cm^−1^ and 761.78 cm^−1^ (δ_C-O_, β-glycosidic linkage, w) evidenced the characteristic nature of a peptidoglycan (Fig. 3C). SC-2 was named ‘rhamnofucosan’ herein due to its unusual high fucose and rhamnose contents. The strong absorption at 280 nm implied the presence of huge amount of aromatic amino acids [32]. The exceptionally large amount of essential amino acid content (80.3%) implicated the traditional medicinal use of SC-2 as a hepatoprotective agent (attributed to cysteine and methionine) and the building blocks for the active sites or signaling sites (usually contributed by tyrosine, cysteine, and histidine) [33] (Table 1).

On the other hand, the apparently perceivable difference in FTIR absorption spectra GmC, SB and SA could be due to the exocyclic methylene between C12 and C13 in both SB and GmC and the benzoyl ester at C6 of GmC. Similar results were reported by Ma et al. [34]. To our astonishment, the FTIR spectra of the pure SC-2 (Fig. 3C) and the lignan+SC-2 appeared extremely alike (Figs. 4A–4C, lower panels), underlying the occurrence of strong intermolecular interaction between lignans and SC-2 due to complete entrapment of lignans into SC-2 macromolecule. Biologically, SC-2 was entirely non-cytotoxic, while the slight decline in viability found for doses ≥800 µgmL^−1^ could have been due to the membrane-masking or -plugging exerted by SC-2 (Fig. 5, right lower panel).

Pharmacokinetically, the uptake rates of both SB and SA were apparently enhanced, conversely, GmC significantly retarded by SC-2 (Table 2). To interpret this, we assumed that the uptake of lignans obeyed first-order kinetics with respect to the free *Schisandra* lignan when used alone (eq. 1), whereas it obeyed a second-order kinetic in the presence of SC-2 (eq. 2):
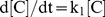
(1)and
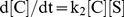
(2)where C is the concentration of SC lignans (µmol L^−1^), t is the duration of incubation time (min), and S is the amount of SC-2 present in the reaction mixture (herein SC-2 = 1 mgmL^−1^ or 1.1891 µmol L^−1^). The parameters k_1_ and k_2_ are first- and the second-order uptake rate coefficients, respectively. The calculated uptake kinetic parameters are listed in Table 2. As shown, in the presence of SC-2, the initial uptake rate constants (in L•mmol^−1^ min^−1^) for SB, GmC and SA were 2.016×10^−5^, 5.046×10^−6^, and 1.264×10^−5^, respectively. A similar trend was also perceivable in free lignans but to a lesser extent (Table 2).

Worth noting, free SA uniquely revealed a relatively delayed uptake rate compared to those of GmC and SA. Intracellular concentrations of GmC were rapidly consumed up at 30 min for GmC and at 60 min for SA, both similarly yielding intracellular decay rates −1.092×10^−6^ L•mmol^−1^ min^−1^ (Table 2), as contrast, the uptake of SB required much longer time. While in the presence of SC-2, the uptake of GmC was significantly suppressed (Table 2). The reason could be attributed to the retardation effect of SC-2 on the benzoyl esteric and C7-OH of GmC (Fig. 1). Over time, the total delivery rates became with the order SB>SA>GmC. Speculatively, the therapeutic indication of whole SC would be mostly depending on SB (Table 2).

Pharmacodynamically, the improved IC_50_ values in time- and dose-dependent manner apparently implied that the enhanced transport of SB (gomisin N) and GmC had been affected by SC-2 (Table 3, Fig. 5A & 5B). A previous report indicated the respective IC_50_ values to be 0.043 and 0.336 mM with respect to human colorectal cancer cell line HT-29 [35]. As contrast, the IC_50_ values for HepG2 cells were 0.19 mM for free SA and 0.15 mM for combined SA with SC-2, indicating cell-specific drug-susceptibilities.

Now the question arises: How did SC-2 affect the pharmacokinetic and pharmacodynamic outcomes of SC-lignans? To solve this issue, the FITC fluorescence technique was applied. Amazingly, labeled SC-2 was shown to have been completely un-mobilized into the cell membrane (Fig. 6A & 6B).

As was described in “Materials and methods”, FITC-SC-2 was added at 0.01, 0.1, 1.0, 10.0, and 25 µgmL^−1^, which respectively corresponded to final concentrations of 0.0025 to 6.25 µgmL^−1^. These amounts elicited approximate coverage rates (number of fmoles of FITC-SC-2 per HepG2 cell) of 3.0×10^−5^ to 7.5×10^−2^ fmoles cell^−1^. Taking the Avogadro's number (6.02×10^23^ molecules/mole) into account, the respective coverage rates became 1.81×10^4^ to 4.52×10^7^ FITC-SC-2 particles/cell, underlying the fuzzy appearance (Fig. 6B). Thus, in order to obtain a clearer image, we adopted concentrations much lower than those used for the MTT assay (Figs. 5A, Fig. 6A, 6B). More importantly, results distinctly revealed SC-2 molecules to be preferentially adhered onto the outer membranes of HepG2 cells (Fig. 6A, 6B), consistent with the widely cited [36], [37]. Literature elsewhere indicated that SA with two methoxy groups respectively located at positions C12 and C13 (Fig. 1) could show the most cytotoxic behavior (i.e. the lowest IC_50_ value) compared to GmC and SB (Gomisin N) [37], [38] (Table 3). The latter two compounds exhibit an exocyclic methylene (-O-CH_2_-O-) linkage instead of two methoxy groups (Fig. 1) [36], [37], indicating exocyclic methylene linking C12 and C13 to be cytotoxicity attenuation-related. Supposedly, the C12 and C13 methoxy groups hindered the SA transport. Conversely, the exocyclic methylene (-O-CH_2_-O-) linkage favored the rapid transport of SB and GmC (Table 2). Strong bioactivity can be attained by lignans structurally without ester group at C-6 and a hydroxyl group at C-7 or an exocyclic methylene chain between C12 and C13, but with an R-biphenyl configuration (Fig. 1, Table 3) [37]. Worth noting, 6(7)-dehydroschisandrol A, a derivative of SA, showed the highest activity (IC_50_, 2.1 µM) as a platelet-activating factor antagonist [38].

SB (Gomisin N) was shown to increase the resistance of mitochondria to calcium ion-induced disruption, effectively preventing the apoptosis of hepatic cells under stressful conditions [39], [40].

TUNEL assay indicated the approximate order of cytotoxicity to be: SA>SB>GmC (Fig. 7A). All the combined therapies elicited rather large extents of apoptosis (Fig. 7B). Interestingly, when treated with combined SC-2 the order of cytotoxicity changed to SC-2+SA>SC-2+GmC>SC-2+SB, consistent with the MTT assay (Table 3).

Now, the question arises “Could such non-spontaneous unidirectional transport be allowed to occur?” To resolve this problem, we performed a theoretical calculation using the Second Law of Thermodynamics (please be referred to Text S1) (Table 4, Table 5). Results in Table 6 indeed evidenced such a “Catcher-Pitcher Unidirectional Transport Mechanism” (Fig. 8, Fig. 9).

Finally, SC-2 exhibited appreciable water solubility (unpublished), implying that the feasible role of decoction in Traditional Chinese Medicinal preparations.

## Conclusions

The pure peptidoglycan SC-2 obtained from *S. chinensis* fruits is nontoxic to the HepG2 cell line. SC-2 increases the transport and cytotoxicity of SC lignans by the “Catcher-Pitcher Unidirectional Transport Mechanism”, underlying the beneficial effect of SC-2 to improve the hepatoprotective effect. Physical chemically, the Second Law of Thermodynamics allows such a unidirectional transport phenomenon. More importantly, the pharmacodynamic behavior greatly improved by the combined therapy (SC-2+lignans) implies the decoction philosophy for preparation of the Traditional Chinese Medicine.

## Supporting Information

Figure S1 HPLC, UV-Visible, ESI-MS and ESI-MS-MS analyses (top to bottom) of dibenzocyclooctadiene lignans isolated from *S. chinensis* fruits. Gomisin C (GmC); deoxyschisandrin (SA); schisandrin B (SB). The molecular weights: gomisin C, 536.6; deoxyschisandrin, 416.5; and schisandrin B, 400.5, respectively.
(TIF)

Text S1 (DOC)
