# Supplementary figures and images for: Schisandra chinensis Peptidoglycan-Assisted Transmembrane Transport of Lignans Uniquely Altered the Pharmacokinetic and Pharmacodynamic Mechanisms in Human HepG2 Cell Model
Source: PLoS One. 2014 Jan 27;9(1):e85165. doi: 10.1371/journal.pone.0085165 (PMC3903492; doi:10.1371/journal.pone.0085165)

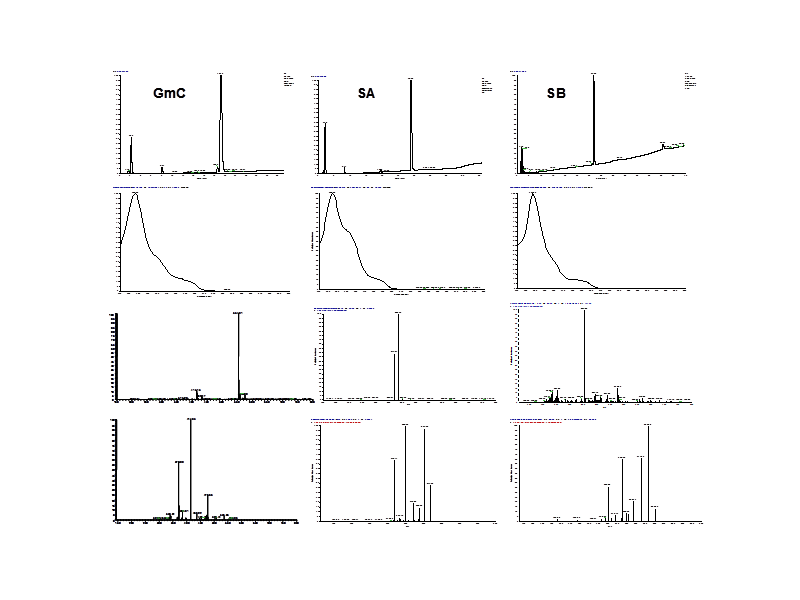

Supplement: Figure S1 — HPLC, UV-Visible, ESI-MS and ESI-MS-MS analyses (top to bottom) of dibenzocyclooctadiene lignans isolated from S. chinensis fruits. Gomisin C (GmC); deoxyschisandrin (SA); schisandrin B (SB). The molecular weights: gomisin C, 536.6; deoxyschisandrin, 416.5; and schisandrin B, 400.5, respectively. (TIF) [file pone.0085165.s001.tif]
