# Supplementary material for: Schisandra chinensis Peptidoglycan-Assisted Transmembrane Transport of Lignans Uniquely Altered the Pharmacokinetic and Pharmacodynamic Mechanisms in Human HepG2 Cell Model
Source: PLoS One. 2014 Jan 27;9(1):e85165. doi: 10.1371/journal.pone.0085165 (PMC3903492; doi:10.1371/journal.pone.0085165)
Supplement: Text S1 — (DOC) [file pone.0085165.s002.doc]

**Supporting Information**

**Text S1**

**1. Isolation and purification of dibenzocyclooctadiene lignans**. Desiccated sample fruits of *S*. *chinensis* were purchased from Sun Ten Pharmaceutical Corp. (Taipei, Taiwan, ROC). Ten grams of desiccated fruits were extracted thrice with 95% of ethanol, each time extracted with 100 ml for 30 min in a sonication-assisted extractor. The extracts were combined and filtered. The filtrate was concentrated by the rotary evaporator to yield 416 mg of the crude extract (yield 4.16%). A semi-preparative HPLC apparatus equipped with a Hitachi (Tokyo, Japan) L-7100 dual pumps connected to a Luna C18 (2) (250 × 10.00 mm i.d.; particle size, 5 mm, Phenomenex, Torrance, CA) column and an L-7455 diode-array detector was used for isolation of each individual compound in the ethanolic extracts at 230 nm (scanning range 220-400 nm). The effluent from column was collected with a fraction collector (Isco Retriever 500, Lincoln NE). The mobile phase operated at a flow rate of 4.0 ml min^−1^ was consisting of two solvents: A and B. Solvent A was 0.1% (v/v) formic acid in HPLC grade water and solvent B was 0.1% formic acid in acetonitrile. The column temperature was maintained at room temperature throughout the operation. The programmed gradient mobile phase was the same as the following HPLC analysis. Sample size of 500 μL for each extract was applied during HPLC analysis. The compounds obtained from this semi-preparative HPLC were concentrated and analyzed using the HPLC-ESI-MS-MS.

**2. Solvent extraction of crude polysaccharides from *S. chinensis****.*

Method for extraction of the crude polysaccharide from SC was carried out according to Ker et al. [25]. Briefly, one hundred grams of pulverized *S*. *chinensis* fruits were firstly refluxed with 2 L of double-distilled water (DDW) at 90 °C with constant stirring at 400 rpm for 2 h. After cooled, the mixture was filtered and the residues was collected. The next steps were conducted as previously cited^36^. Finally, the final sediment was collected, dialyzed, and lyophilized to recover the isoelectric precipitate (SC crude polysaccharide, SC-CP; yield 7.24%).

**3. Purification of SC-CP**. Further isolation and purification of SC-CP were conducted with the gel permeation chromatography (GPC) was carried out according to Ker et al. [25]. For quantification, the phenol-sulfuric acid colorimetric method of Dubois et al. was followed for monitoring the content of carbohydrate and peptido moiety [26, 31]. The optical density (OD) was read at 490 nm against the control. Otherwise, the absorbance of the crude polysaccharides SC-CP eluents obtained from GPC was directly read at 280 nm at ambient temperature for determination of protein content. Bovine serum albumin (BSA) was used as the reference standard (linearity range: 0.001-0.063 mg/mL; Y=0.0787X-0.084, R^2^ = 0.991). While the carbohydrate content was assayed using glucose as the reference standard (linearity range: 0.001-0.500 mg ml^−1^; Y=8.9667X-0.1654, R^2^ = 0.998) [25]. For more accurate measurement of molecular weight, the separated fraction numbers 33 to 39 in GPC were collected, concentrated (SC-2) and further characterized with high performance size exclusion chromatography-tandem UV-Vis and evaporative light scattering detection (HPSEC-UV-ELSD) analysis.

**4. Characterization of the molecular weight and the molar extinction coefficient with HPSEC-UV-ELSD**. The high performance size exclusion chromatography-tandem UV-Vis and evaporative light scattering detection (HPSEC-UV-ELSD) analysis was conducted to determine the molecular weight of SC-2. We described the detailed method in Supplementary Methods.

The polysaccharides (10 mg) collected from fraction No. 33-39 were dissolved in water (1 mL) and was analyzed at 280 nm with the Hitachi LC-7150 system (Hitachi, Tokyo, Japan) coupled with an UV-Vis detector (SPDA-6AV, Shimadzu, Japan) and an evaporative light scattering detector (Sephadex 75, Seder Corporation, France). A Phenomenex pre-column polySep-GFC-P (75×7.8 mm) connected to PolySep-GPC-P 4000 column (300×7.8 mm, Phenomenex, Torrance, CA, USA) was warmed up to 40°C. The sample solution (20 μL) was injected. The isocratic elution was started with double distilled water at a flow-rate 0.8 mL/min. The parameters of ELSD were set as follows: The drift tube temperature, 50 °C; and the nebulizer nitrogen gas pressure, 2.3 bar. The molecular mass distribution and related mean molecular mass were determined by comparing with a standard curve established by the authentic pullulans (Sephadex standard P-82 kit, Showa Denko K. K., Tokyo, Japan) having known molecular masses 5.9, 11.8, 22.8, 47.3, 112, 212, 404 and 788 kDa, respectively. The average molecular mass was calculated from the linear correlation (ℓogDa = -0.467X +10.023, R² = 0.9937). Where Da is the logarithm of the authentic molecular mass and X is the retention time (min) of each authentic pullulan.

**5. X-ray powder diffraction (Powder XRD) of SC-2**. The desiccated purified SC-2 powder was macerated to fine and homogenous powder and subjected to X-Ray powder diffraction analyzer (X'Pert Pro MRD, PANalytical B. V., Almelo, The Netherlands). The powder was filled into the sample holder having a volume 1 cm^3^, compactly and evenly. The sample stage configuration was PW3071/xx bracket. The scan axis used was Gonio. The goniometer PW3050/60 Theta was operated at a minimum step size of 2 theta = 0.001. The minimum step size omega was 0.001. The diffractometer system used was XPERT-PRO. The anode metal was Cu target that emitted K_α_ source with wavelengths of K_α1_ and K_α2_ to be 1.540598 Å and 1.544426 Å, respectively. The ratio of K_α1_ was 0.5. The divergence was fixed at 1.52 mm. The monochromator used was NO. The generator volt was 45 KV. The tube current was 40 mAmp. The uni cell was specified at hkl = 000. The scan range was set at 2θ = 5^o^C to 2θ = 80^o^C. The san speed was set at 0.5 deg-min^-1^ with scan steps set at 0.033423 deg. The number of point taken was 2244. The scan type was operated at “continuous”. The time per step was set at 149.86.
**6. Monosaccharide composition in SC-2**.

The method for analysis of monosaccharide composition was according to previous work of Ker et al.^35^ Briefly, SC-2 was subjected to hydrolysis, and the liberated sugars were transformed into alditol acetates as previously reported with a slight modification^35^. The final residue was dissolved in 1 ml acetone. An aliquot (1 μl) of which was injected into the Gas Chromatograph–Mass Spectrometer (GC–MS) apparatus and analyzed as cited [25]. The identification and quantification of monosaccharides were carried out by comparing the spectra with those of authentic standards. The peak areas of all identified sugar compounds were quantified and expressed in mole percentage.

**7. Amino acid composition in the protein moiety of SC-2**.

The method for analysis of amino acid composition was according to previous work of Ker et al. [25]. Briefly, standard amino acid solution (0.3 mL of authentic sample solution) and 0.6 mL of SC-2 were transferred into 3 ml reaction vessels. To which 0.01 ml of internal standard norleucine solution (10 mg ml^−1^) was added. The following protocol was conducted as cited [25]. Finally, the dehydrated chloroform extract obtained was applied to GC-MS analysis by conditions similar to that for monosaccharide analysis.

**8. Source and cell lines stock**. The human hepatocellular carcinoma cell line HepG2 (BCRC 60380) and the BALB/c mouse embryonic normal hepatic cell line (BNL CL.2 cells) was obtained from the Bioresource Collection and Research Center (BCRC, Food Industry Research and Development Institute, Hsin-Chu City, Taiwan). The cell lines were incubated at 37 ^o^C under a 5% CO_2_ atmosphere in DMEM supplemented with serum. Each 500 mL of which had been previously reinforced with 10% of heat-inactivated FBS, 1% of antibiotics (penicillin 100 IU and streptomycin 100 μg) and 50 μL of 2 mM glutamine before inoculation.
**9. Cell culture and cell viability assay**.

HepG2 cells were cultured in DMEM containing 10% heat-inactivated FBS, 100 units/mL of penicillin, and 100μg/mL of streptomycin at 37 °C in 5% CO_2_. The culture medium was changed fresh twice a week and the cells were subcultured at a 1:4 ratio once a week. The tetrazolium dye (3-(4,5-dimethylthiazol-2-yl)-2,5-diphenyltetrazolium bromide, MTT) colorimetric assay was conducted following the manufacturer’s instruction. Briefly, HepG2 cells were cultured in a 96-well microplate at a cell density 1.5×10^5^/mL for 24 h. The cells were rinsed twice using PBS and treated with different concentrations of purified lignans in the presence of absence of SC-2 as indicated. After 24 h incubation, MTT assay was carried out. The absorbance was measured at 570 nm, from which the percentage viability was calculated.

**10. Determination of the uptake rate of free schisandrin B, gomisin C, and deoxyschisandrin in the absence and the presence of SC-2 by HepG2 cells**.

HepG2 cells (5×10^5^ cells /mL) were seeded onto six 10 cm dishes (3 dishes A1, A2, and A3; and 3 dishes B1, B2, and B3) as reported previously^39^ with a slight modification. After the HepG2 cells were cultured in DMEM (containing 10% heat-inactivated FBS, 100 units/mL of penicillin, and 100 μg/mL of streptomycin) at 37 °C in 5% CO_2_ for 5 days, the supernatant was removed. The cells were rinsed thrice with PBS. To dishes A1, A2 and A3 0.1 mM of samples schisandrin B, gomisin C and deoxyschisandrin were added respectively; to dishes B1, B2, and B3 0.1 mM of samples schisandrin B, gomisin C and deoxyschisandrin, each containing 1 mg/mL of SC-2, were added respectively. The incubation was continued for 5, 15, 30 and 60 min. The supernatant was removed and the cells were rinsed thrice with PBS. To all dishes trypsin-EDTA was added and incubated for 5 min at 37°C in 5% CO_2_. The cells were harvested at 17000×g for 10 min. To the cells 500 μL cell lysis buffer were added and the mixture was left to stand for 30 min. the lyzed cells were centrifuged at 17000×g for 10 min. To the supernatant obtained 1 mL of methanol was added. After concentrated to 500 μL, aliquots 20 µL of the samples were subjected to HPLC analysis. The HPLC was equipped as above mentioned: HPLC Pump, Hitachi 5110; the column, Phenomenex Luna C18 3µm (i.d. 150×2 mm); Detector, Hitachi L7455 monitoring at 240 nm. The mobile phase used consisted of two parts: the mobile phase A was HPLC grade water containing 0.1% formic acid. The mobile phase B was acetonitrile containing 0.1% formic acid. The gradient elution was programmed as follows: from 0-30 min solvent A 70% + solvent B 30%; form 30-40 min, solvent A 5% + solvent B 95%; from 40-50 min, solvent A 5% + solvent B 95%; from 50-70 min, solvent A 70% + solvent B 30%; and from 70 min on, solvent A 70% + solvent B 30% was used. The flow rate was operated at 0.2 mL/min.

**11. Determination of the intracellular disposition of SC-2 in HepG2 cells.**

**11.1. Fluorescein isothiocyanate (FITC) labeling of SC-2.**

FITC-labeled polysaccharides SC-2 (FITC-SC-2) were prepared according to Kanebo et al. [28] and Tanaka et al. [29] with slight modification. Briefly, 100 mg (118.9 nmoles) of SC-2 was dissolved in 10 mL PBS solution containing 50 μL of pyridine, 40 μg of FITC (102.7 nmoles) and 2 mg of dibutyltin dilaurate. The labeling reaction was carried out at 95 °C for 2 h. After repeated precipitations with ethanol (1:4, v/v), the FITC-labeled SC-2 was dialyzed through dialysis bags (Seamless cellulose tubing, small size No. 24, Wako, Osaka, Japan) for 96 h and then lyophilized.
**11.2. Intracellular disposition of FITC-labeled SC-2.**

The HepG2 cells (1×10^5^ cells/mL) were seeded onto a 3.5 cm dish containing 2 mL of DMEM medium. After incubated for 24 h at 37 °C, FITC-SC-2 at 0.01, 0.1, 1.0, 10.0, and 25 μg/mL were added and incubated for 30 min to see the dose-affected effect. Alternatively to see whether the incubation time could affect the disposition, the HepG2 cells (1×10^5^ cells/mL) were seeded onto a 3.5 cm plate containing 2 mL DMEM and incubated for 24 h at 37 °C, FITC-SC-2 (10.0 μg/mL) was added and incubated at 37°C for 5, 15, 30 and 60 min, respectively. At each set point, 1 μL-aliquots of the cells were withdrawn with a micropipette and the cells were rinsed four times with ice-cold PBS and examined under the IX-71 Olympus inverted fluorescence microscope (Olympus, Tokyo, Japan). The microphotographs were taken with the attached 35 mm single-lensed reflex camera (DP-72, Olympus).

**12. TUNEL assay.**

The TUNEL assay using the Fluorescein Apoptosis Detection Kits (Roche Applied Science, PA, USA) was carried out according to the manufacturer’s instructions and Borisov et al. [30]. Briefly, HepG2 cells (3×10^3^ cells /mL) were seeded onto 10 cm dishes and cultured in DMEM (containing 10% heat-inactivated FBS, 100 units/mL of penicillin, and 100 μg/mL of streptom) at 37 °C under 5% CO_2_ atmosphere for 24 h to serve respectively the medium control, SC-2 control, three free lignans, and SC-2 plus three lignans. The PI staining and TUNEL assay were carried out according to Gavrieli et al. [31] DNA fragments were labeled with the In Situ Fluorescein Apoptosis Detection Kits. Thus, both the apoptotic and the non-apoptotic cells can be stained red by propidium iodide, whereas fluoresecin-12-dUTP will be ‘nicked” at the 3’-OH ends of fragmented DNA [31], to yield green fluorescence localized within the nuclei of apoptotic cells. Images were examined with the IX71 inverted fluorescence microscope (Olympus, Tokyo, Japan).

**13. Theoretical Deduction by “The Second Law of Thermodynamics”**

In order to interpret the unique unidirectional transport of lignans in the presence of SC-2 that actually is allowed by “The Second Law of Thermodynamics”, we performed the following theoretical calculation adopting the kinetic data obtained in this experiment.

Table 4 indicates the extracellular concentration of Schisandrin B was 0.007 mM, and astonishingly the intracellular concentration already had reached 0.093 mM. Obviously, such a mass transport phenomenon is completely against the passive transport rule. Instead, SC-2 was able to elicit active transport, even though the intracellular Gomisin C and deoxyschisandrin concentrations were much lower than the extracellular (Table 4). The Second Law of Thermodynamics allows the unidirectional forward transport to occur spontaneously under condition with a rather large net negative free energy change. Supposedly SC-2 can play a role to direct such a unidirectional transport (Fig. 8 & Fig. 9). Based on the accumulating semi-empirical findings, we developed a diagrammatic model (Fig. 8). The change of concentration profile during the transport of lignans in the absence and the presence of SC-2 is shown in Fig. 8. Worth noting, SC-2 alone did not have any effect on HepG2 cells (Fig. 5 & 6). However, in the presence of SC-2 huge improvement in transport of free lignans spontaneously occurred at 37^o^C (Table 2 & Table 4). The common passive transport in the absence of SC-2 is shown as Path 1 (Fig. 9). Thus as hypothesized, in an isothermal transport system at 37^o^C involving SC-2 coexisting with the HepG2 cells and SC lignans, SC-2 tends to rapidly bind up with the outer membrane (Fig. 8 & 9, Eq. 3), simultaneously with a conformational change to commit a decreased free energy change (ΔG_0,1_ << 0) (Eq. 3).

…….........3

For which the equilibrium constant

$$\text{K}_{\text{eq1}}\text{=[SC-2-M}_{\text{outer}}\text{]/[SC-2][M}_{\text{outer}}\text{] >>1}$$

$\text{=K}_{3}\text{/K}_{3}^{\text{,}}$ ……………………………………………………………4

In reality, as [M_outer_] >>> [SC-2], the concentration of [M_outer_] could be considered to be a “constant” denoted as K. Hence Eq. 4 is modified as

$\text{K}_{\text{eq1}}^{\text{,}}\text{=K}_{\text{eq1}\bullet}\text{K=[SC-2-M}_{\text{outer}}\text{]/[SC-2]}$ ...............................................5

The free energy change is

$\text{ΔG}_{\text{0,1}}\text{= -RT }l\mathrm{n}\text{K}_{\text{eq1}}^{,}$ …..………………………………………….….…6

Where R = -8.314 9 J K^-1^mol^-1^; T = 310 K. Eq. 3 must have fulfilled K_eq1_’>>1 to give the free energy change ∆G_0,1_<< 0. Only under such a condition, the equilibrium could greatly favor the forward reaction and SC-2 tends to overwhelmingly cover the outer membrane in form of [SC-2-M_outer_] conjugate once having become contacted with the cell membrane (Eq. 3, Fig. 8 & 9). Expectedly, SC-2 particles would be more concentrated at the bulk fluid side of outer membrane (Fig. 8 & 9). Moreover, the accommodation of new conformation elicited by SC-2-membrane conjugates would be more specifically to accumulate the SC lignans (SCLs or Ls) (the “Catcher Transport” named herein) according to Eq. 7:

………............…….…7

Where $\text{K}_{\text{eq2}}\text{=[SC-2-M}_{\text{outer}}\text{Ls]/[SC-2-M}_{\text{outer}}\text{] /[Ls]}$

$\text{=K}_{4}\text{/K}_{4}^{\text{,}}\text{ }>>1$ ……………………………............………...…8

And $\text{ΔG}_{\text{0,2}}\text{= (-RT }l\mathrm{n}\text{K}_{\text{eq2}}\text{) <<0}$ …………………......…......………………..9

Due to the rapid depletion of SCLs by the “Catcher Transport” at the junction of outer membrane, the concentration of SCLs in bulk fluid immediately adjacent to the outer membrane would have rapidly declined to C_om_ (Fig. 9, Eq. 7), which actually obeys the transport equation (Eq. 10):

$\text{(dC/dx)=k}_{4}\text{(C}_{0}\text{-C}_{\text{om}}\text{)}$ ……………………………………………….…...10

where k_4_ is the mass transfer coefficient of SCLs from the bulk fluid to the SC-2-M_outer_ complex in Path 2 (Fig. 9). By such a SC-2 mediated unidirectional transport, the free SC lignans would be easily mobilized into the intramembrane compartment once they dissociate from the complex SC-2-M_outer_-L_s_ (Eq. 11, Fig. 9). As a consequence, the intramembrane concentration of Ls could be instantaneously elevated high up to a level C_mA_, and subsequently dropped to C’_mA_ due to the inner membrane resistance, implicating the pseudo-active transport path 2 (with SC-2) to be more effective than the passive transport path 1 (without SC-2) (Fig. 9).

……...…...11

Worth amazing, the second step in Path 2 proceeds against a concentration gradient from C_om_ to C_mA_ (Fig. 9) whose free energy change ∆G_0,3_ is positively far greater than zero (Eq. 11 and Eq.12), i.e.

$\text{ΔG}_{\text{0,3}}\text{= (-RT }l\text{n}\mathrm{C}_{\text{om}}\text{/C}_{\text{mA}}\text{) >>0}$ …………………………...………………..12

In reality this step (Eq. 12) would be thermodynamically unfavorable, when the lignans (Ls) were released from SC-2-M_outer_-Ls conjugates as Ls_in_ into the cell cytoplasm (Eq. 11). The intracellular peak concentrations of Schisandrin B and Deoxyschisandrin would be instantaneously elevated (conversely that of Gomisin C decreased due to its C6 esteric and C7 hydroxyl interactions), which in fact already had been verified in present experiment (Table 2). As seen, the peak concentration rapidly reached 91 µM at 60 min and 77 µM at 15 min, respectively, while that of Gomisin C was inhibited to only 19 µM at 15 min (Table 2). Comparing to the free lignas in the absence of SC-2, the corresponding peak levels could have only reached 82 µM and 63 µM for Schisandrin B and Deoxyschisandrin respectively, but up to 27 µM for Gomisin C (Table 2).

The third stage mass transport may spontaneously occur in the cytoplasm with the condition

$\text{(dC/dx)=k}_{6}\text{(C}^{\text{,}}_{\text{mA}}-\mathrm{C}_{\text{mE}}\text{)}$ ……………………………..…………………13

However, the intracellular degradation occurred in the cytoplasm at a rate -1.092×10^-6^ L•mmol^-1^min^-1^ (Table 2), there could not be a true equilibrium occurring under such a circumstance. Alternatively, the pseudoequilibrium theory can be applied, i.e.

$$\text{ΔG=G}_{\text{state 2}}\text{- G}_{\text{state 1}}$$

$\text{=[G}_{\text{state 2}}\text{(C}_{2}\text{)]-[G}_{\text{state 1}}\text{(C}_{1}\text{)]}$ .......................................................................14

$=\text{-RT }l\text{nC}^{\text{,}}_{\text{mA}}\text{/C}_{\text{mE}}$ .......................................................................................15

Since C’_mA_ >> C_mE_, (Fig. 9, Table 4) the corresponding free energy change would become greatly negative in magnitude.

$\text{ΔG}_{\text{0,4}}\approx\text{(-RT }l\text{nC}^{\text{,}}_{\text{mA}}\text{/C}_{\text{mE}}\text{) <<0}$ …………………………………….…..16

The significantly raised intracellular concentration of schisandrin B and deoxyschisandrin in the presence of SC-2 (Table 2) apparently has verified the condition that the overall free energy change in Path 2 of these two lignans in reality has reached highly negative in magnitude, i.e.

$\Delta G_{\text{overall}}=(\Delta G_{0,1}+\Delta G_{0,2}+\Delta G_{0,3}+\Delta G_{0,4})<<0$ …………………………17

Alternatively, in the presence of SC-2 Gomisin C had a positive ∆G_0,3_ value to give a more positive ∆G_overall_. Speculatively, Gomisin C possesses a hydroxyl group at position C7 which can extraordinarily bind with SC-2 (Fig. 1), resulting in more difficult releasing of Ls into the cytoplasm (Eq. 18).

……….………18

In a dynamic transport process like the one we are facing, the true equilibrium condition in reality could not be attained at all. Instead, we were only able to evaluate the parameters related with the so-called “Pseudoequilibrium”. Thus from the experimental analysis we in reality could only obtain parameters related to such a “Pseudoequilibrium”-linked transport (Table 4), and these parameters were used for calculation of the relevant free energy changes (Table 5). Eventually, we arrived at the overall free energy change ΔG_overall_ = -<< 0 for all SC-2 mediated SC-lignan transports (Table 6). To our knowledge, this is the first report addressing such a fantastic and unique “Catcher and Pitcher Unidirectional Transport Mechanism” of SC-2.

**Supplementary Figure legend**

**Fig. S1.** The HPLC chromatograms, UV spectra, MS and MS/MS spectra (order in top-down) of GmC, SA and SB, respectively.
